# Reduction of dose to duodenum with a refined delineation method of Para-aortic region in patients with locally advanced cervical Cancer receiving prophylactic extended-field radiotherapy

**DOI:** 10.1186/s13014-019-1398-6

**Published:** 2019-11-08

**Authors:** Bo Yang, Xiaoliang Liu, Ke Hu, Jie Qiu, Fuquan Zhang, Xiaorong Hou, Junfang Yan, Qingyu Meng, Weiping Wang, Lang Yu, Yijun Wang

**Affiliations:** Department of Radiation Oncology, Peking Union Medical College Hospital, Chinese Academy of Medical Sciences & Peking Union Medical College, NO.1 Shuaifuyuan Wangfujing, Dongcheng District, Beijing, People’s Republic of China 100730

**Keywords:** Cervical cancer, Prophylactic extended field radiotherapy, Target delineation, Dosimetric comparison

## Abstract

**Background:**

To compare irradiation dose to the second and third portions of duodenum (Duo2 and Duo3) with a new refined and old delineation method of para-aortic region for patients with locally advanced cervical cancer (LACC) receiving prophylactic extended-field radiotherapy (EFRT).

**Methods:**

Twenty consecutive patients with LACC were treated with prophylactic EFRT from January 2016 to January 2017 at our institute. Two delineation methods of para-aortic region were designed for each patient, the old delineation method ensured a full coverage of aortic and inferior vena cava, while the right paracaval region above L3 was omitted from CTV in the new delineation method. Patients received a dose of 50.4Gy in 28 fractions for PCTV and a dose of 60.2Gy in 28 fractions for PGTV with volumetric-modulated arc therapy (VMRT). The dose delivered to Duo2 and Duo3 with these two delineation methods were compared.

**Results:**

All treatment plans achieved excellent target volume coverage with 95% of PCTV receiving 50.4Gy and 95% of PGTV receiving 60.2Gy. There was no difference between delineation methods in low dose level (V5, V10, V15, V20, V25) for Duo2 and Duo3. The V30, V35, V40, V45, V50, Dmax, Dmean and D2cc for Duo2 with the new and old delineation methods were 55.76% vs 80.54% (*P* = 0.009), 34.72% vs 70.91% (*P* < 0.001), 18.69% vs 55.46% (*P* < 0.001), 8.20% vs 41.49% (*P* < 0.001), 1.86% vs 21.60% (*P* < 0.001), 49.58Gy vs 52.91Gy (*P* = 0.002), 30.38Gy vs 39.22Gy (*P* = 0.001) and 37.90Gy vs 48.64Gy (*P* < 0.001) respectively. For Duo3, the new delineation method achieved significant advantages in V30, V35, V40, V45, V50 and Dmean over the old one (96.82% vs 99.25%, *P* = 0.021; 89.65% vs 97.21%, *P* = 0.001; 79.50% vs 93.18%, *P* < 0.001; 65.63% vs 82.93%, *P* < 0.001; 43.39% vs 65.60%, *P* < 0.001; 46.09Gy vs 49.24Gy, *P* < 0.001), no deference was observed regarding D2cc and Dmax with these two delineation methods.

**Conclusion:**

With the new delineation method of para-aortic area in prophylactic EFRT, significant reduction of irradiation dose to the second and third portions of duodenum in high dose area was obtained. This may further lower the incidence of duodenal toxicity when performing prophylactic EFRT for patients with LACC.

## Background

Para-aortic lymph node metastasis (PALNM) significantly impairs the prognosis of patients with cervical cancer [1]. It was reported that the frequency of PALNM varied from 16 to 25% for patients with locally advanced cervical cancer (LACC) [2]. Para-aortic lymph nodes are usually evaluated by imaging methods. A meta-analysis showed that the sensitivities for compute tomography (CT), magnetic resonance imaging (MRI), and positron emission tomography/CT (PET/CT) in the detection of positive lymph nodes were 50, 56, and 82%, respectively [3]. Therefore, a proportion of microscopic positive para-aortic lymph nodes may be missed with these imaging modalities.

Prophylactic extended-field radiotherapy (EFRT) has been used for patients with LACC with high risk of PALNM. Even though some patients with LACC could get survival benefits from prophylactic EFRT, the incidence of treatment related toxicities were still high due to large radiation field and large volumes of organs at risk [4–6]. Duodenum is adjacent to the para-aortic region and portions of the organ are immobile, especially for the second portion (descending) and the third portion (transverse) [7]. All these factors may expose the duodenum to high dose irradiation and cause high incidence of radiation related toxicities when performing EFRT. A study from MD Anderson cancer center found that the 3-year actuarial rate of any duodenal toxicity was 11.7% for patients with gynecologic cancer receiving para-aortic lymph nodes irradiation [8].

Recently, several studies demonstrated that the distribution of PALN was asymmetrical and very few lymph nodes were in the right paracaval region (RPC), especially above the third lumbar vertebra (L3, 9–11]. Thus, the RPC region above the level of L3 may potentially be omitted from clinical target volume (CTV) when contouring the para-aortic lymph nodes region. With this method of delineation, dose to duodenum may also be potentially decreased.

To identify this probability, we conducted a dosmetric study to compare dose to the duodenum with this new refined and old contouring methods of para-aortic region when performing prophylactic EFRT for patients with LACC.

## Methods

From January 2016 to January 2017, twenty consecutive patients with biopsy proven cervical cancer who received prophylactic extended-field radiotherapy due to positive common iliac lymph nodes, were selected into this dosimetric study.

Before treatment, patients were simulated under supine, head-first position with abdominopelvic CT (16-slice Philips Brilliance Big Bore CT) in a 5-mm slice thickness. Thermoplastics were used to immobilize patients, and full bladder, empty rectum, vaginal markers were also prepared for patients before simulation.

**Fig. 1:**
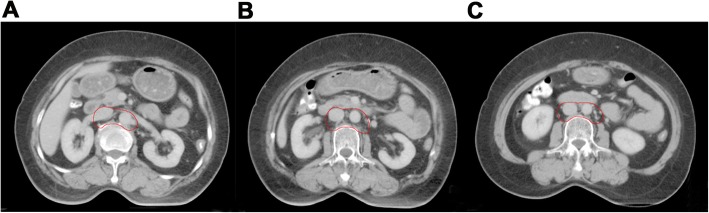
**a-c** The old delineation method of para-aortic region (The aorta and inferior vena cava area should be fully covered by CTV)

**Fig. 2:**
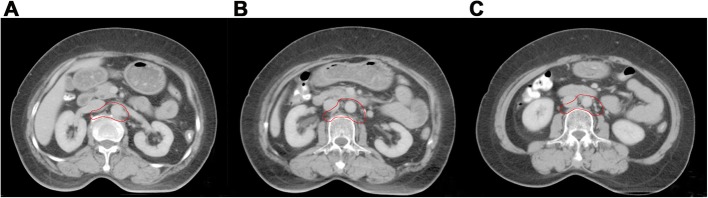
**a-c** The new delineation method of para-aortic region (The anterior and left regions of IVC above the level of L3 was omitted from the CTV)

All patients received definitive chemoradiotherapy in this study. Clinical target volume (CTV) included pelvic and para-aortic areas. Delineation of the pelvic area included primary tumor, cervix, parametrium, uterus, vagina (depending on the extent of primary tumor) and pelvic lymphatic drainage area (common iliac, external iliac, internal iliac, obturator and presacral lymph nodes). Para-aortic region was contoured according to two methods. In the first method, para-aortic region contouring was based on method proposed by Keenan LG [9] (shown in Table 1, Fig. 1a-c) ensuring a full coverage of aorta and inferior vena cava (IVC). While in the second method, the anterior and left regions of IVC above the level of L3 was omitted from the CTV (Fig. 2a-c). The upper border of CTV in both methods was at the L1/L2 space. CTV plus a 7-10 mm margin was defined as planning clinical target volume (PCTV). Gross tumor volume (GTV) covered the positive pelvic lymph nodes. A 5 mm margin was added to GTV to form planning gross tumor volume (PGTV). A dose of 50.4Gy was prescribed to at least 95% of PCTV with intensity modulated radiotherapy (IMRT). At least 95% of PGTV was escalated to 60.2Gy with simultaneous integrated boost (SIB) technique. A total dose of 30-36Gy in 5–7 fractions was administrated to point A with Ir-192 resource. Cisplatin (30-40 mg/m2, peer week) was the first line regimen for concurrent chemotherapy.

**Table 1:** The old delineation method for para-aortic area in prophylactic EFRT [9]

Steps	Methods
1	Contour the inferior vena cava and aorta
2	The superior extent should be the at the L1/L2 space
3	The inferior extent should be the level of the bifurcation of the aorta.
4	Expand the aorta by a margin of 10 mm anteriorly, posteriorly and medially, and 15 mm laterally.
5	Expand the inferior vena cava by 8 mm anteriorly and medially, and 6 mm posteriorly and laterally.
6	Combine the aorta and inferior vena cava expansions to create a clinical target volume.
7	Crop the clinical target volume form normal boundaries such as the vertebral body, muscle and bowl and expand the posterior border to the anterior vertebral body.

*Abbreviations*: *EFRT* extended-field radiotherapy

**Fig. 3:**
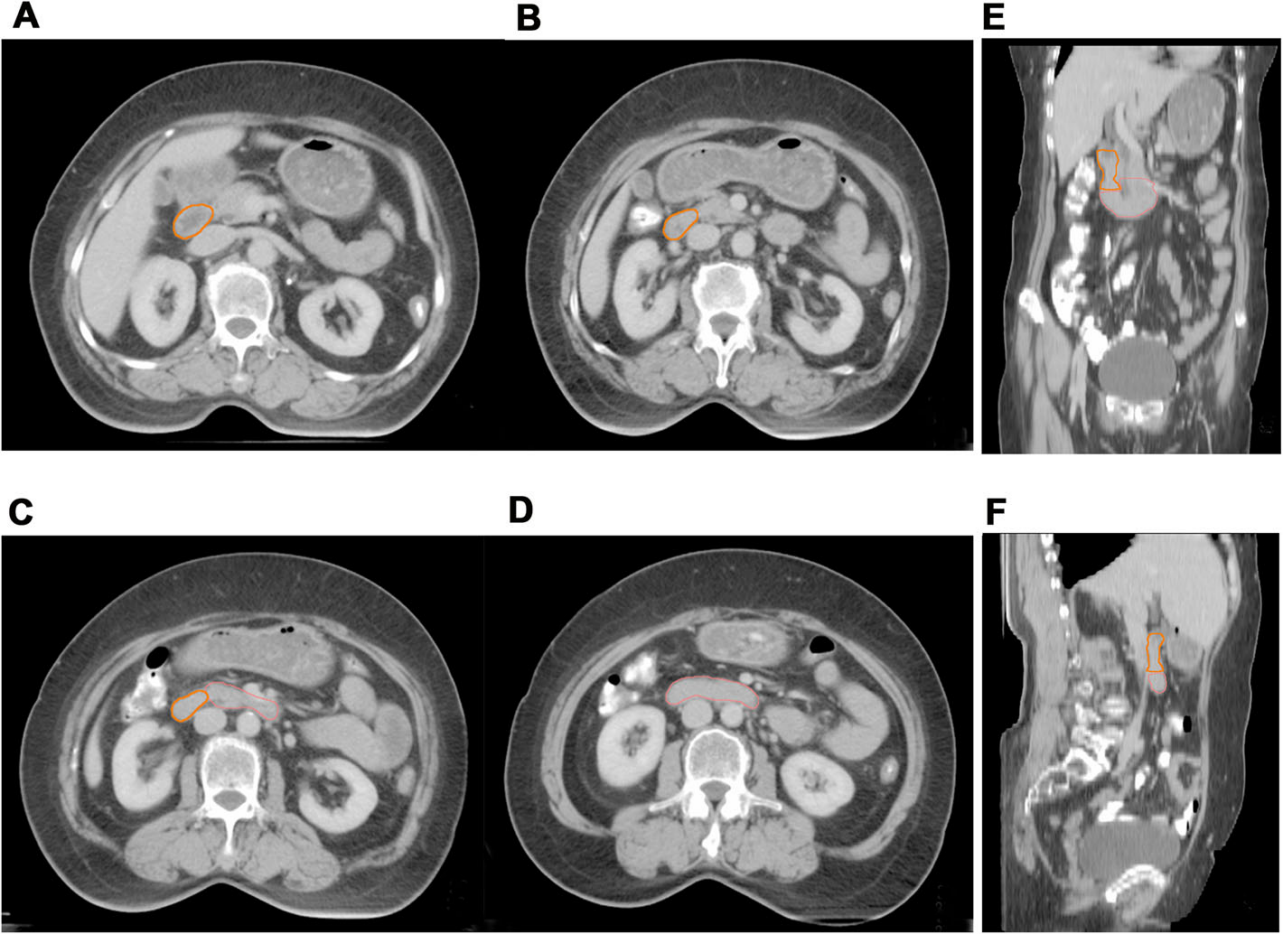
**a-f** The delineation of the second and third portions of duodenum (Duo2 and Duo3). (The orange line represented for Duo2; the pink line represented for Duo3)

Small intestine, kidneys, bladder, spinal cord and femoral heads were routinely outlined as organs at risk (OARs). Delineation of the second (descending Duo2) and third (transverse Duo3) portions of duodenum referred to guidelines proposed by Radiation Therapy Oncology Group (RTOG) (Fig. 3a-f) [10]. The dose constrains of OARs were as follows: spinal cord, D0.1cc ≤ 45Gy; small intestine, V25 ≤ 50%; D2cc ≤ 54Gy; bladder, V45 ≤ 50%; rectum, V45 ≤ 50%; kidneys, V20 ≤ 30%; Duo2, Dmax≤53Gy; Duo3, Dmax≤53Gy.

Volumetric-modulated arc therapy (VMAT) treatment plans was performed with Eclipse Version 8.6 system. Two arc fields (179°-181°, 181°-179°) with 6-MV X photon were designed for VMAT. We designed two VMAT treatment plans for each patient based on two delineation methods mentioned above. We generated dose volume histograms (DVH) of each delineation methods from treatment plans. The dose delivered to Duo2 and Duo3 with these two delineation methods were compared.

All enrolled patients were treated with the new refined delineation method. The first follow-up examination was performed 1 month after treatment. Then, patients received follow-up examination every 3 months in the first 2 years, every 6 months in the next three to 5 years and once a year thereafter. Routine follow-up examinations were suggested in our previous study [11]. Gastrointestinal endoscopy was not routinely recommended.

The differences between doses to OARs of different delineation methods were compared with Student’s t test using SPSS 23.0 software. A two side *P* value of < 0.05 was defined as statistically significant.

## Results

### Dosimetric comparison

**Fig. 4:**
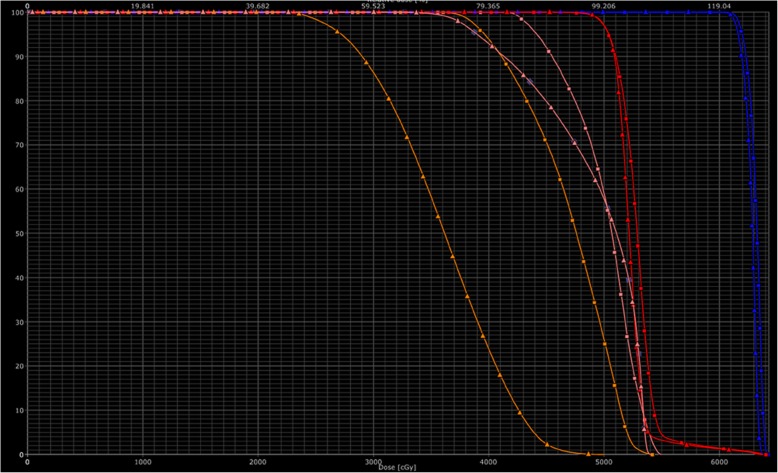
Comparation of the dose volume histograms (DVH) with the two delineation methods for a patient with cervical cancer receiving prophylactic extended-field radiotherapy (blue line-PGTV, red line-PCTV, orange line-Duo2, pink line-Duo3, quadrate box-old delineation method, triangle box-new delineation method)

All treatment plans achieved excellent target volume coverage with 95% of PCTV receiving 50.4Gy and 95% of PGTV receiving 60.2Gy. We compared volumes of Duo2 and Duo3 receiving doses at each 5Gy interval with the two delineation methods. Comparation of the dose volume histograms (DVH) with the two delineation methods for a representable patient is shown in Fig. 4.

For Duo2 (shown in Table 2), there was no difference between delineation methods in low dose level (V5, V10, V15, V20, V25). When it comes to dose level of 30Gy or higher, significant reductions were observed at each 5Gy interval with new delineation method compared with the old one. The mean volumes of Duo2 receiving dose of 30Gy (V30), 35Gy (V35), 40Gy (V40), 45Gy (V45) and 50Gy (V50) with the new and old delineation methods were 55.76% vs 80.54% (*P* = 0.009), 34.72% vs 70.91% (*P* < 0.001), 18.69% vs 55.46% (*P* < 0.001), 8.20% vs 41.49% (*P* < 0.001), 1.86% vs 21.60% (*P* < 0.001), respectively. The maximal dose (Dmax), mean dose (Dmean) to Duo2 and dose to 2cm^3^ (D2cc) of Duo2 were also significant reduced by 6.3, 22.5 and 22.1% with the new delineation method (49.58Gy vs 52.91Gy, *P* = 0.002; 30.38Gy vs 39.22Gy, *P* = 0.004; 37.90Gy vs 48.64Gy, *P* < 0.001).

**Table 2:** Volumes of Duo2 receiving irradiation dose at each 5 Gy interval with the two delineation methods

	Old delineation method (Mean ± Std %)	New delineation method (Mean ± Std %)	P
V5	100%	99.70 ± 1.04%	0.211
V10	95.77 ± 9.11%	94.47 ± 11.48%	0.695
V15	92.58 ± 13.60%	88.55 ± 17.02%	0.413
V20	89.56 ± 16.40%	81.53 ± 22.08%	0.200
V25	86.17 ± 19.19%	71.37 ± 29.11%	0.065
V30	80.54 ± 23.51%	55.76 ± 32.76%	**0.009**
V35	70.91 ± 27.91%	34.72 ± 26.06%	**< 0.001**
V40	55.46 ± 29.79%	18.69 ± 17.94%	**< 0.001**
V45	41.49 ± 24.06%	8.20 ± 10.77%	**< 0.001**
V50	21.60 ± 16.83%	1.86 ± 3.13%	**< 0.001**
Dmean	39.22 ± 8.31Gy	30.38 ± 7.98Gy	**0.001**
Dmax	52.91 ± 1.45Gy	49.58 ± 4.14Gy	**0.002**
D2cc	48.64 ± 4.10Gy	37.90 ± 6.90Gy	**< 0.001**

*Abbreviations*: *Duo2* the second portion of duodenum, *Dmean* the mean irradiation dose to Duo2, *Dmax* the maximal irradiation dose to Duo2

*P* < 0.05 values are statistically significant

For Duo3 (shown in Table 3), no difference was found in volumes receiving less than 30Gy at each 5Gy interval (V5, V10, V15, V20, V25) between the two delineation methods. However, the new delineation method achieved significant advantage in volumes receiving dose of 30Gy (V30) compared with the old one (96.82% vs 99.25%, *P* = 0.021). Similar advantages of V35 (89.65% vs 97.21%, *P* = 0.001), V40 (79.50% vs 93.18%, *P* < 0.001), V45 (65.63% vs 82.93%, *P* < 0.001) and V50 (43.39% vs 65.60%, *P* < 0.001) were observed with the new delineation method. Compared with old delineation method, the mean dose to Duo3 (Dmean) was reduced by 6.4% with new delineation method (46.09Gy vs 49.24Gy vs, *P* < 0.001). There was no significant difference regarding Dmax and D2cc between the two delineation methods. No volume of Duo2 and Duo3 received dose of more than 55Gy in both delineation methods.

**Table 3:** Volumes of Duo3 receiving irradiation dose at each 5 Gy interval with the two delineation methods

	Old delineation method (Mean ± Std %)	New delineation method (Mean ± Std %)	P
V5	100%	100%	–
V10	100%	100%	–
V15	100%	100%	–
V20	100%	100%	–
V25	99.98 ± 0.09%	99.70 ± 0.80%	0.122
V30	99.25 ± 1.63%	96.82 ± 4.23%	**0.021**
V35	97.21 ± 3.93%	89.65 ± 8.58%	**0.001**
V40	93.18 ± 6.53%	79.50 ± 11.99%	**< 0.001**
V45	82.93 ± 15.64%	65.63 ± 14.25%	**< 0.001**
V50	65.60 ± 11.40%	43.39 ± 14.30%	**< 0.001**
Dmean	49.24 ± 1.60Gy	46.09 ± 2.55Gy	**< 0.001**
Dmax	53.96 ± 2.15Gy	53.69 ± 1.10Gy	0.634
D2cc	52.60 ± 0.89	52.38 ± 0.75	0.392

*Abbreviations*: *Duo3* the third portion of duodenum, *Dmean* the mean irradiation dose to Duo3, *Dmax* the maximal irradiation dose to Duo3

*P* < 0.05 values are statistically significant

### Clinical outcomes

The median follow-up duration was 26.5 months (range: 10.2–43.4 months). Three patients suffered disease relapse. One patient had supraclavicular and mediastinal lymph node metastasis, the other one experienced cervical lymph node metastasis, the last one suffered pelvic recurrence and brain metastasis. No patient experienced para-aortic lymph node recurrence.

The incidence of acute and chronic gastrointestinal (GI) toxicity is listed in Table 4. Most of the acute gastrointestinal toxicities were recoverable. The incidence of grade 3 nausea and diarrhea were 20 and 20%, a total of five patients (25%) experienced grade 3 acute GI toxicity. As for chronic GI toxicity, only one patient suffered grade 3 proctitis.

**Table 4:** The incidence of acute and chronic GI toxicity

Toxicity	Acute			Chronic		
	G1	G2	G3	G1	G2	G3
Nausea	7 (35%)	3 (15%)	4 (20%)	1 (5%)	2 (10%)	0 (0)
Abdominal pain	7 (35%)	2 (10%)	0 (0)	3 (15%)	0 (0)	0 (0)
Diarrhea	7 (35%)	1 (5%)	4 (20%)	4 (20%)	1 (5%)	0 (0)
Proctitis	3 (15%)	1 (5%)	0 (0)	1 (5%)	4 (20%)	1 (5%)
Total	11 (55%)	4 (20%)	5 (25%)	4 (20%)	5 (25%)	1 (5%)

*Abbreviation*: *GI* gastrointestinal

## Discussion

Several studies have identified the potential benefit of EFRT for patients with LACC [4–6]. A randomized controlled trial (RCT) from Sandi Arabia showed that patients with LACC receiving extended-field concurrent chemoradiotherapy had superior survival outcomes than those receiving pelvic concurrent chemoradiotherapy only [6]. In our retrospective study [5], patients with EFRT had significant decreased 3-year distant failure rate and 3-year para-aortic lymph node failure rates compared with patients receiving pelvic RT (7.0% vs 21.7%, *P* = 0.016; 0% vs 6.6%, *P* = 0.014). A tendency of improved DFS was also observed with EFRT (80.6% vs 71.0%, *P* = 0.097). Another prospective study is conducting to compare the efficacy and tolerance between EFRT and pelvic radiotherapy in selected patients with LACC at our institute (NCT03955367). Though the survival outcomes of prophylactic EFRT for patients with LACC were promising, we should not ignore radiation related toxicities arising by large radiation field of EFRT.

The reported acute and late incidence of grade 3 or greater (G3+) gastrointestinal toxicity for cervical cancer patients receiving prophylactic EFRT varied largely from 4 to 20% [5, 12–14]. Duodenum is the beginning of the small intestine, which is adjacent to para-aortic area. The second and third parts of duodenum are usually immobile [7]. All these characteristics make duodenum more easily be exposed to high dose irradiation, and more vulnerable than other parts of small intestine. Verma, J and colleagues reported a 3-year actuarial duodenal toxicity rate of 11.7% when performing EFRT for patients with gynecological cancer [8]. Therefore, controlling duodenal toxicities is of great difficulty and importance for EFRT, especially when with prophylactic intent.

With the development of physical technology, IMRT has already been widely used in the treatment of gynecological cancer. The advantages of IMRT in decreasing dose to OARs and reducing radiation related toxicities have also been proved by lots of researches [15–17]. However, approximate 5% of patients with LACC still suffered G3+ gastrointestinal toxicity after EF-IMRT [5]. Another potential method to limit dose exposure to normal tissues and toxicities might be refining the CTV, which means omitting unnecessary volumes of CTV.

Three studies were conducted to determine the distribution of para-aortic lymph nodes for the sake of more accurate contouring of para-aortic lymph node area [9, 18, 19]. Kabolizadeh P, et al. enrolled 46 patients with gynecological cancer, one hundred thirty-three positive PALNs were identified with imaging modalities. Seventy-eight positive lymph nodes (59%) were in the left PA region (between aorta and left psoas muscle, LPA) and 47 lymph nodes (35%) were in the aortocaval region (between inferior vena cava and aorta, AC), only eight lymph nodes (6%) were in the right paracaval region (between inferior vena cava and right psoas muscle, RPC). Another interesting finding was that all malignant PALNs were inferior to or at the level of renal vessels. Takiar and colleagues from MD Anderson Cancer Center got similar conclusions with Kabolizadeh P [19], the majority of positive PALNs on Positron Emission Tomography/Computed Tomography (PET/CT) were located in the LPA (37/72, 51.4%) and AC (32/72, 44.4%) regions, the RPC region only contained three malignant lymph nodes (3/72, 4.2%). They further measured the distance between the aortic bifurcation and the top of T12 vertebral body and divided the para-aortic area into three parts (superior, middle, inferior). Notably, all positive lymph nodes of the RPC region were limited to the inferior third of para-aortic region, at or below L3, and within 3 cm of the aortic bifurcation. Keenan LG, et al. [9] also concluded that all identified positive PALNs were located inferior to left renal vein, and no RPC lymph node was above L2. Based on these three distribution maps of PALNs, irradiating the upper area of the RPC region might result in unnecessary dose to nearby normal tissues, especially duodenum. It is reasonable to omit the RPC region above L3 from CTV when performing prophylactic EFRT.

In this dosimetric study, we depicted two delineation methods of para-aortic region and compared the irradiation doses to the second and third portions of duodenum (Duo2, Duo3) between these two delineation methods. No differences were observed regarding V5, V10, V15, V20, V25 of Duo2 and Duo3. With the new delineation method, the V30, V35, V40, V45, V50, Dmean, Dmax and D2cc of Duo2 were significantly reduced by 30.8, 51.0, 66.3, 80.2, 91.4, 22.5, 6.3 and 22.1%, respectively. The V30, V35, V40, V45, V50, Dmean of Duo3 with the new delineation method were significantly lower than those with the old delineation method.

Clinical and dosimetric studies have revealed that V55 was a relevant factor for duodenal toxicities [8, 20]. Kelly P, et al. retrospectively reviewed 106 patients with locally advanced unresectable pancreatic cancer (LAPC), all patients received neoadjuvant and concurrent chemotherapy. Seventy-eight patients received a prescribed dose of 50.4Gy in 28 fractions and 28 patients were treated with dose escalation of 57.5–75.4Gy in 28–39 fractions. Multivariate analysis showed significant relationship between V55 and duodenal toxicity. Patients with V55 ≥ 1 cm^3^ suffered more G2+ duodenal toxicity than those with V55 < 1 cm^3^ (47% vs 9%, *P* = 0.0003) [20]. Another study from Verma J, et al. assessed duodenal toxicity in 105 patients with gynecologic cancer with positive PALNs. Patients with V55 ≥ 15 cm3 suffered higher 3-year actuarial rate of duodenal rate than those with V55 < 15 cm3 (48.6% vs 7.4%, *P* < 0.01) [8]. In our study, no volume of Duo2 and Duo3 received more than 55Gy irradiation dose, because we prescribed a relatively low dose of 50.4Gy in 28 fractions to para-aortic area for prophylactic intent, while most patients received a dose of more than 55Gy in the studies mentioned before. Except for V55, there is a tendency of increasing G2+ duodenal toxicity with the increase of V45, V50 and Dmean, the 1-year incidence of G2+ duodenal toxicity for patients with V50 above and below 25cm^3^ were 22 and 11%, respectively (*P* = 0.13, 20]. With the new para-aortic delineation method we suggested, significant reduction of V30, V35, V40, V45, V50 and Dmean for Duo2 and Duo3 were observed compared with the old delineation method. We have enough rationale to speculate that this new delineation method would be helpful in limiting duodenal toxicity for patients with LACC receiving prophylactic EFRT.

Considering the anatomical relationship between duodenum and CTV, prophylactic EFRT in LACC is different from radiotherapy in pancreatic cancer and definitive EFRT in LACC. For patients with pancreatic cancer receiving radiotherapy, the whole duodenum is adjacent to CTV [8]. When performing definitive EFRT, the upper border of CTV is usually at the interspace of T12/L1 [11]. In these cases, regarding four parts of duodenum as a single organ is more reasonable. While the upper border of CTV in prophylactic EFRT is just at the interspace of L1/L2, the first and fourth portions of duodenum are far away from CTV, irradiation dose to these two portions is quite limited. Therefore, evaluating dose to the second and third parts of duodenum is of more significance.

As far as we know, this is the first study regarding irradiation dose to duodenum for patients with LACC receiving prophylactic EFRT. We innovatively proposed a new refined delineation method of para-aortic area based on distribution maps of para-aortic lymph nodes which is quite convincible. A significant reduction of volumes of both Duo2 and Duo3 in high dose region was obtained with the new delineation method. However, we have to admit that our study still leaves much to be desired. Though we reported gastrointestinal toxicities in this study, it is hard to identify duodenal toxicity without gastrointestinal endoscopy examination. At present, we couldn’t perform gastrointestinal endoscopy examination for patients, since it isn’t routinely recommended during patients’ follow-up. Lacking in clinical record about duodenal toxicity, we couldn’t further corelate dosimetric data with incidence of duodenal toxicity. Prospective randomized controlled study is needed to confirm the advantage of this new delineation method over the old one.

## Conclusion

With the new delineation method of para-aortic area in prophylactic EFRT, significant reduction of irradiation dose to the second and third portions of duodenum in high dose area was obtained. This may further lower the incidence of duodenal toxicity when performing EFRT for patients with LACC.

## Data Availability

The datasets used and analyzed during the current study are available from the corresponding author on reasonable request.

## Abbreviations

CT: Compute tomography
CTV: Clinical target volume
EFRT: Extended field radiotherapy
GTV: Gross tumor volume
IMRT: Intensity modulated radiotherapy
LACC: Locally advanced cervical cancer
MRI: Magnetic resonance imaging
OAR: Organs at risk
PALNM: Para-aortic lymph node metastasis
PCTV: Planning clinical target volume
PET/CT: Positron emission tomography/compute tomography
PGTV: Planning gross tumor volume
